# 46,XY Disorder of Sex Development Caused by 17*α*-Hydroxylase/17,20-Lyase Deficiency due to Homozygous Mutation of* CYP17A1* Gene: Consequences of Late Diagnosis

**DOI:** 10.1155/2018/2086861

**Published:** 2018-04-24

**Authors:** Giampaolo Papi, Rosa Maria Paragliola, Paola Concolino, Carlo Di Donato, Alfredo Pontecorvi, Salvatore Maria Corsello

**Affiliations:** ^1^Endocrinology Unit of the Northern Area, Azienda USL di Modena, Modena, Italy; ^2^Unit of Endocrinology, Università Cattolica del Sacro Cuore and Fondazione Policlinico Universitario Agostino Gemelli, Rome, Italy; ^3^Laboratory of Molecular Biology, Institute of Biochemistry and Clinical Biochemistry, Catholic University of Sacred Heart, Rome, Italy; ^4^Department of Internal Medicine, Azienda USL Modena, Modena, Italy

## Abstract

**Context:**

Congenital adrenal hyperplasia (CAH) is an autosomal recessive disease due to specific enzyme deficiencies in the adrenal steroidogenesis pathway.

**Case Description:**

A 40-year-old Chinese woman was referred to the Endocrine Unit for the work-up of a syndrome characterized by long-lasting and multidrug resistant high blood pressure, severe hypokalemia with metabolic alkalosis, and primary amenorrhea. The patient presented with sexual infantilism, lack of breast development, absence of axillary and pubic hair, tall stature, and slenderness. CT scan revealed enlarged adrenal glands bilaterally and the absence of the uterus, the ovaries, and the Fallopian tubes. Furthermore, diffuse osteopenia and osteoporosis and incomplete ossification of the growth plate cartilages were demonstrated. Chromosomal analysis showed a normal male 46,XY, karyotype, and on molecular analysis of the* CYP17A1* gene she resulted homozygous for the g.4869T>A; g.4871delC (p.Y329Kfs?) mutation in exon 6. Hydrocortisone and ethinyl-estradiol supplementation therapy led to incomplete withdrawal of antihypertensive drug and breast development progression to Tanner stage B2 and slight height increase, respectively.

**Conclusions:**

We describe a late-discovered case of CAH with 46,XY disorder of sex development. Deficiency of 17*α*-hydroxylase/17,20-lyase due to a homozygous CYP17A1 gene mutation was the underlying cause. Laboratory, imaging, and genetic features are herein reported and discussed.

## 1. Introduction

Congenital adrenal hyperplasia (CAH) is an autosomal recessive disease due to specific enzyme deficiencies in the adrenal steroidogenesis pathway [1]. Depending on either the type of enzyme mutation or the level of enzymatic activity, the clinical presentation of CAH is manifold [2]. Whatever be the underlying enzyme mutation, the biochemical result is characterized by the impairment in cortisol biosynthesis.

21-Hydroxylase deficiency is by far the most frequent cause of CAH, accounting for approximately 95% of CAH forms, and is caused by mutations in the gene encoding for a cytochrome P450* (CYP21A2)* [3]. Mutations in the cytochrome P450 family 17 subfamily A member 1* (CYP17A1)* gene located on chromosome 10q24.3 lead to the rare deficiency of 17*α*-hydroxylase/17,20-lyase [4]. The clinical features of such a severe CAH subtype are the consequence of the enzymatic block at the level of the pregnenolone's and progesterone's conversion towards glucocorticoid and sex steroid production [5]. Indeed, this combined enzyme deficiency affects both the adrenal and the gonadal steroidogenesis. The accumulation of mineralocorticoid precursors (mainly, deoxycorticosterone and corticosterone) causes hypokalemic alkalosis and hypertension. The impaired gonadal steroidogenesis causes a lack of androgens in males and, then, a 46,XY disorder of sex development with variable phenotypes including a completely female phenotype. Also, a lack of estrogen in both males and females occurs, provoking a primary amenorrhea in 46,XX and a low bone mineral density in both sexes.

To date, several mutations in the* CYP17A1* gene have been reported in the literature [5–11]. The most severe deficiency affects both steps (17*α*-hydroxylase and 17,20-lyase) of the enzyme's activity, whereas, in even the rarest cases of mild deficiency, 17*α*-hydroxylase activity can be preserved leading to isolated 17,20-lyase deficiency with only gonadal deficiency.

Here, we describe the peculiar clinical, laboratory, imaging, and genetic features of a Chinese patient affected by 17*α*-hydroxylase/17,20-lyase deficiency.

## 2. Case Report

A 40-year-old Chinese woman was admitted to the Emergency Room because of a serious car crash. She had received concussion, traumatic rupture of the aortic isthmus, and multiple fractures to left femur, left elbow, right leg, and pelvis. She underwent endovascular aortic stent graft and fractures' closed reduction and immobilization in a plaster cast. Although the head computed tomography (CT) revealed a tentorial blood suffusion, the patient did not experience neurologic damage. Lab-analysis showed the following: normal renal, hepatic, and thyroid function; normal coagulation tests; normal white cell and platelet count; mild normocytic anemia (Hb = 10 g/dl); metabolic alkalosis associated with severe hypokalemia.

Patient's past history consisted of long-lasting high blood pressure and primary amenorrhea. She was born in China (Zhejiang), where she had been living for 28 years; later, she was married and moved to Carpi (Modena), Italy, where she was employed in a textile factory. Since her twenties, she was taking a multidrug antihypertensive and potassium replacement therapy, which on admission included doxazosin 8 mg/day, lisinopril 20 mg/day, hydrochlorothiazide 25 mg/day, amlodipine 10 mg/day, canrenone 100 mg/day, bisoprolol 5 mg/day, and potassium chloride 1800 mg/day. Furthermore, owing to the recent implantation of aortic stent graft, she was given acetylsalicylic acid 100 mg/day. The patient declared that all attempts to get pregnant failed and, therefore, she had adopted a male kid. She stated that she was the eldest of three siblings: a 36-year-old sister, who was taking antihypertensive drugs, suffered of primary amenorrhea, and was sterile; a 34-year-old sister, who was mother of 3 kids and was doing well. Her father had died because of lung carcinoma (maybe due to heavy smoking), whilst her mother was alive and was doing well. The two sisters and the mother still live in China.

Once the patient's clinical conditions improved, she was referred to the Endocrine Unit, Department of Internal Medicine, for the endocrine work-up.

The patient presented with sexual infantilism, lack of breast development, complete absence of axillary and pubic hair, tall stature, and slenderness. The external genitalia were phenotypically female, the height was 175 cm, the weight was 64 Kg, and the body mass index (BMI) was 20.9. On admission, the blood pressure (BP) was persistently high throughout the day: mean systolic BP values were 170 mmHg; mean diastolic BP values were 110 mmHg. The patient received biochemical and endocrine lab tests. The results—which are summarized in Table 1—showed the following: low-undetectable levels of testosterone, DHEA-S, androstenedione, 17-OH-progesterone, estradiol and active renin; low-normal 24-h urinary cortisol and serum aldosterone; high ACTH, progesterone, LH and FSH concentrations; metabolic alkalosis with severe hypokalemia. The clinical evidence and laboratory results both suggested the diagnosis of the rarest forms of CAH rather than the commonest 21-hydroxylase deficiency. Chromosomal analysis demonstrated a normal male 46,XY, karyotype. Interestingly, when the patient's sex life was examined, despite the prepuberal condition, she acknowledged having weekly—albeit unsatisfying (i.e., anorgasmic)—intercourses.

**Figure 1:**
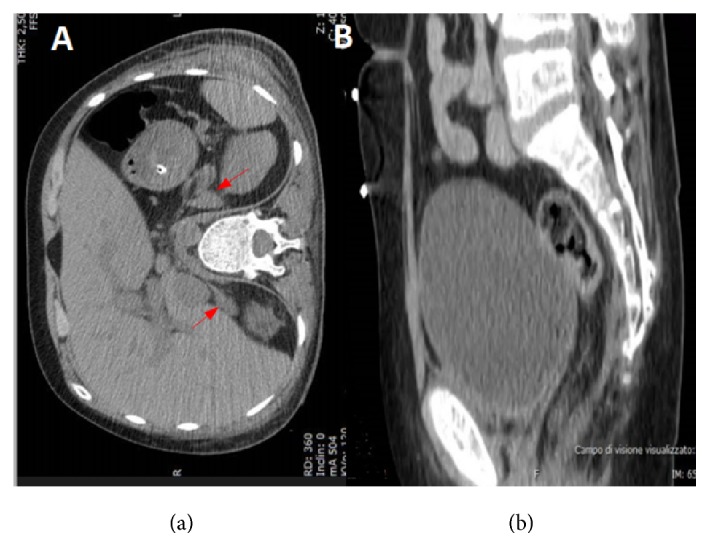
Abdomen and pelvis CT scan. (a) The adrenal glands are enlarged bilaterally (red arrow); (b) gonads are not detectable.

**Figure 2:**
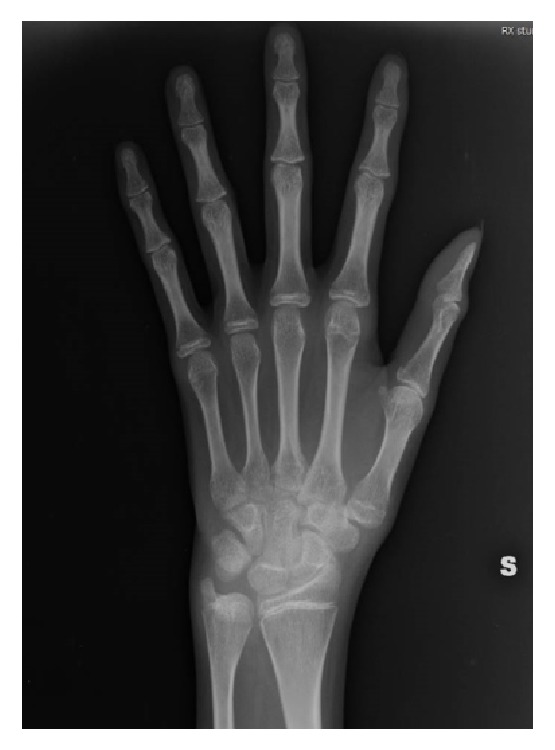
Left hand and wrist X-ray. The ossification of the epiphysial cartilage of distal end of radius and ulna, 1st metacarpus, and phalanxes is not yet complete.

A computed tomography of the abdomen and pelvis showed enlarged adrenal glands bilaterally (Figure 1(a)); the absence of the uterus, the ovaries, and the Fallopian tubes was shown, too (Figure 1(b)). Furthermore, the radiography of the left hand and wrist revealed that the ossification of the epiphysial cartilage of distal end of radius and ulna, 1st metacarpus, and phalanxes was not yet complete (Figure 2). To investigate the possible negative impact of chronic hypoestrogenic state, the bone mineral density (BMD) was checked at the lumbar spine and the femoral neck, and it was consistent with osteoporosis and osteopenia, respectively. In particular, the *T*-score and the *Z*-score at L1–L4 level were reduced at −3.6 SD and −3.4 SD, respectively; they were equal to −2.4 SD and – 2.2 SD at the femur, respectively.

**Figure 3:**
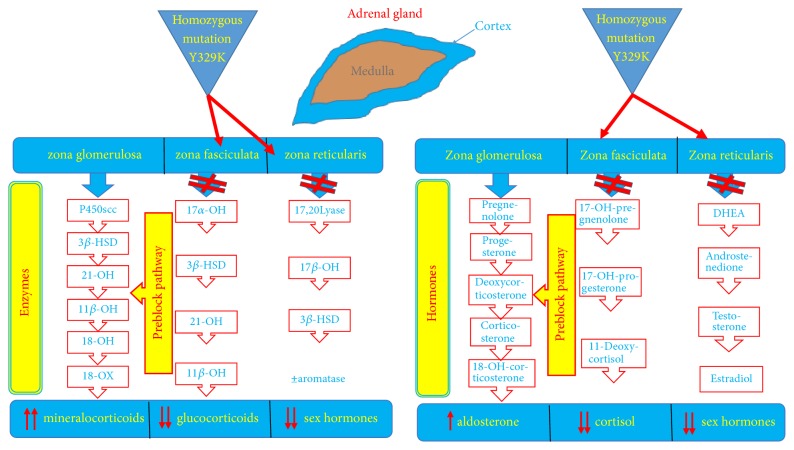
The adrenal steroid biosynthetic pathway: involved enzymes, hormones, and precursors. The “block” sites of hormone production in patients affected by 17*α*-hydroxylase/17,20-lyase deficiency are highlighted by the red stop signs.

Overall, the clinical, laboratory, and imaging features were consistent with CAH caused by combined 17*α*-hydroxylase and 17,20-lyase deficiency, as suggested by very high progesterone levels and low 17-OH-progesterone and sex steroids values (Figure 3). To confirm this diagnosis, the molecular analysis of the* CYP17A1 *gene was performed as previously reported [12].

Mutation analysis by direct DNA sequencing demonstrated the base change from T to A at position 4869 (g.4869T>A) and the deletion of the g.4871 nucleotide (g.4871delC): such genetic alterations lead to the substitution from tyrosine to lysine at aminoacidic position 329 producing a frameshift (p.Y329K*fs*?). The patient resulted homozygous for the most prevalent* CYP17A1* mutation in Chinese patients [13]. A possible loss of heterozygosity (LOH) was excluded using the SALSA MLPA probemix P334-A3 Gonadal Development Disorder (MRC Holland) in a MLPA (Multiplex Ligation-dependent Probe Amplification) assay.

Treatment with oral hydrocortisone 20 mg/day and ethinyl-estradiol 1 mg was started. One month later, step by step, all the antihypertensive drugs—with the only exception of amlodipine—and potassium supplements could be stopped, since blood pressure values constantly were in the normal range. Six months after estradiol supplementation, the patient's breast development progressed to Tanner stage B2 and her stature grew to 177 cm.

Table 1 summarizes the results of biochemical and endocrine analysis performed before patient's discharge from the hospital.

The patient signed the written informed consent for both genetic examinations and use of personal data in the current study.

## 3. Discussion

The present report describes a CAH patient with 46,XY disorder of sex development due to cytochrome P450c17 deficiency caused by the homozygous g.4869T>A; g.4871delC (p.Y329Kfs?) mutation in exon 6 of* CYP17A1 *gene. The P450c17 deficiency is very rare per se, representing almost 1% of all CAH cases, with an estimated incidence of 1 in 50,000–100,000 individuals [13, 14]. What is peculiar to this case and makes it unique lies on the following: (i) the remarkable delay in the diagnosis of CAH and, nonetheless, the possibility of investigating the long-term consequences of P450c17 enzyme deficiency on the clinical point of view; (ii) the combined loss of 17*α*-hydroxylase and 17,20-lyase action; (iii) the homozygous mutation in* CYP17A1 *gene associated with male XY karyotype.

P450c17 is a cytochrome that plays a crucial role in the adrenal and gonadal steroid biosynthetic pathway (Figure 3). Indeed, it carries out two enzymatic activities: the 17-hydroxylase and the 17,20-lyase activity. The first one acts by converting pregnenolone in to 17OH-pregnenolone and progesterone into 17OH-progesterone; the 17,20-lyase activity converts 17OH-pregnenolone into dehydroepiandrosterone and, to a lesser extent, 17OH-progesterone into androstenedione [15–17]. The final result is the block of production of cortisol and sex steroids and the accumulation of preblock metabolites of the mineralocorticoid pathway (mainly, 11-deoxycorticosterone, corticosterone, and 18-OH-corticosterone), overstimulated by ACTH hypersecretion due to the attempt by the hypophysis gland to solve the cortisol deficiency. In this context, severe hypokalemic alkalosis and high blood pressure develop, owing to the potent mineralocorticoid effects of aldosterone precursors, which induce on the one hand sodium and fluid retention and on the other hand loss of potassium and hydrogen. Thus, the renin-angiotensin-aldosterone system is shut down, and laboratory analyses detect suppressed renin and low-normal aldosterone values. It should be noted that the low cortisol production is balanced by the high concentrations of corticosterone, which exhibits a mild glucocorticoid effect [18, 19].

The same attempt made by the corticotroph cells to overcome cortisol deficiency is (unsuccessfully) performed by the gonadotroph cells, which produce high amounts of luteinizing (LH) and follicle-stimulating (FSH) hormone in response to undetectable sex hormone levels. The impairment of sex hormone production leads to a manifold spectrum of clinical manifestations, ranging from mildest forms—characterized by infertility with phenotype corresponding to karyotype—to severe forms presenting with ambiguous genitalia or disorders of sex development [20]. In our patient, the altered biosynthetic process manifested clinically with lack of secondary sexual characteristics (including complete absence of axillary and pubic hair, primary amenorrhea, and no breast development), tall stature, and high blood pressure. Besides obvious hyperplasia of the adrenal cortex, imaging exams did detect neither the female (uterus, the ovaries, and the Fallopian tubes) nor the male (prostate, seminal vesicles, and vas deferens) genitalia. This picture confirms that both the 17-hydroxylase and the 17,20-lyase activity are essential for gonadal steroidogenesis [21].

With respect to high blood pressure values detected on admission, the glucocorticoid substitution therapy allowed the partial discontinuation of antihypertensive drugs. In most patients suffering from 17-hydroxylase/17,20-lyase deficiency reported in the literature [5, 8], the glucocorticoid supplementation was able to maintain normal blood pressure values after complete discontinuation of antihypertensive drugs. We suppose that, in our patient, long-standing hypertension caused irreversible damage of the cardiovascular system. This hypothesis was supported by echocardiophic exam, showing left ventricle hypertrophy (data not shown).

A further interesting finding in this case was the lack of ossification of the epiphysial cartilages, as demonstrated by X-ray of the wrist. Such a feature is the consequence of lacking action of sex hormones on cartilage and bone maturation. The same mechanism explains the important reduction in bone mineral density found in our patient. Thus, a double repercussion occurs on the clinical side: the patient presents with a tall stature and is still growing up; she has a significantly fragile and osteoporotic bone. Actually, except anecdotal cases [10], most Chinese subjects affected by 17-hydroxylase/17,20-lyase deficiency are unusually tall with respect to their ethnicity. The bone mineral density in our patient was frankly low compared to subjects of the same age; probably, both an earlier diagnosis of the underlying disease and the prompt sex hormone substitution therapy would have prevented the development of osteopenia or, at least, of osteoporosis. With regard to the best sex hormone substitution therapy, since our patient was assigned the female sex and had always considered herself as female, we suggested her taking estradiol. Of interest, according to previous observations [5], at 6-month follow-up she experienced breast development and she was satisfied and grateful for this. Moreover, she was 2 cm taller than before starting therapy.

In conclusion, we report a case of 17*α*-hydroxylase/17,20-lyase deficiency causing CAH with 46,XY disorder of sex development. Although further research occurs, this work sheds new light on role of cytochrome P450c17 in the steroidogenesis at both the adrenal and the gonadal level and on the harmful consequences of its deficiency in the short and long term, respectively.

## Figures and Tables

**Table 1:** Results of biochemical and endocrine tests in the patient reported, before and after the administration of estradiol and glucocorticoid therapy.

Lab test	Results		Normal range
	*Before treatment*	*After therapy*	
*TSH* (mcIU/ml)	2.4	NA	0.35–4
*ACTH* (pg/ml)	655.7	19.3	4.3–52
*Active renin* (mcIU/ml)	<0.5	<0.5	4.4–46.1
*Aldosterone* (pg/ml)	23.1	26	30–150
*24-h urinary cortisol* (mcg/24 h)	60	304	58–403
*FSH* (mIU/ml)	40.8	0.8	1.5–12.4
*LH* (mIU/ml)	23.7	0.7	1.8–12
*Estradiol* (pg/ml)	<10	NA	10–40
*DHEA-S* (mcg/ml)	0.04	0.05	0.48–2.44
*Progesterone* (ng/ml)	6.5	NA	0.2–1.4
*17-OH-progesterone* (ng/ml)	0.2	NA	0.2–1.3
*Androstenedione* (ng/dl)	<10	<10	85–275
*Testosterone* (ng/ml)	<0.1	<0.1	2.4–9.5
*pH* (venous sample)	7.56	7.4	7.37–7.45
*Na+* (mEq/L)	140	136	136–146
*K+* (mEq/L)	2.1	4.1	3.5–5.3

*ACTH* = adrenocorticotrophic hormone; *DHEA-S* = dehydroepiandrosterone sulfate; *FSH* = follicle-stimulating hormone; *LH* = luteinizing hormone; *NA* = not available; *TSH* = thyroid stimulating hormone.
